# Particle Deposition in Drying Porous Media

**DOI:** 10.3390/ma14185120

**Published:** 2021-09-07

**Authors:** Emmanuel Keita

**Affiliations:** Navier Laboratory, University Gustave Eiffel, ENPC, CNRS, 77420 Champs-sur-Marne, France; emmanuel.keita@univ-eiffel.fr

**Keywords:** porous media, water transfer, nanoparticles, drying

## Abstract

The drying of porous media is a ubiquitous phenomenon in soils and building materials. The fluid often contains suspended particles. Particle deposition may modify significantly the final material, as it could be pollutants or clogging the pores, decreasing the porosity, such as in salt, in which particles and drying kinetics are coupled. Here, we used SEM and X-ray microtomography to investigate the dried porous media initially saturated by nanoparticle suspensions. As the suspensions were dried, nanoparticles formed a solid deposit, which added to the initial solid matrix and decreased the porosity. We demonstrate that since the drying occurred through the top surface, the deposit is not uniform as a function of depth. Indeed, the particles were advected by the liquid flow toward the evaporative surface; the deposit was significant over a depth that depended on the initial volume fraction, but the pore size was affected over a very narrow length. These findings were interpreted in the frame of a physical model. This study may help to design better porous media and take into account particle influence in drying processes.

## 1. Introduction

The drying of porous media is a ubiquitous phenomenon. The liquid is rarely pure in most environments, but some particles are suspended, and salts are dissolved. The migration of colloids in soil and their deposition are significant for pollutant transport and soil structuration [1,2,3,4]. For building materials and cultural heritage stones, salt may deteriorate the matrix or lead to carbonation of the structures [5,6,7].

Many studies described the evaporation of pure water from porous media with micrometric pores. The general process of drying/drainage is driven by capillary forces [8,9,10,11]. Drying does not occur in a dry area extending progressively from the free surface. However, the capillary effect has been shown to play a major role in water distribution and drying kinetics [12,13]. The evaporated water at the free surface is replaced by liquid flow.

Initially dissolved in water, salt concentrates and eventually crystallizes. The salt concentration is usually higher close to the free surface, and salt may be transported by the fluid flow [14,15]. Moreover, supersaturation is needed for crystallization [16]; even in simple geometry, the crystal localization is complex. Moreover, salt crystals may block further evaporation or provide a porous path for liquid water to favor evaporation [17,18]. Usually, salt crystals form close to the evaporative surface, where salt concentration increases [19,20]. It appears that salt fills the pore partially; after drying, the pore structure and the porous media proprieties are modified [21,22,23].

Due to drying, particle volume fraction increases, and the solid deposit, submitted to stresses, may crack [24,25]. In porous media, colloids may adsorb onto surfaces [26,27,28,29,30,31]. The particle deposit itself is a porous media that may crack and drain water [32,33]. However, particle suspensions have similarities with ionic solutions. Particles are advected by flow [34,35]. However, particles deposit does not require a change of state. Particles are deposited close to the evaporative surface but also distribute inside the sample as the air/water interface recedes in the porous media [36,37,38,39]. The drying kinetics is much slower than for pure water. Colloids’ transport and interaction with surfaces have been studied for saturated porous media; the fixation of colloids to the solid matrix is usually due to surface chemistry [40,41,42]. As water evaporates, suspended particles will eventually form a solid deposit in the porous structure. Large colloid deposits modify the porosity, which changes permeability [43] and thus water flow [44]. However, the description of the dried porous media and the consequences on further transport properties have not been assessed.

In this paper, we observed, by scanning electron microscopy (SEM) and X-ray microtomography, dried porous media initially saturated by nanoparticle suspensions. As the suspensions are fully dried, nanoparticles form a solid deposit, which is added to the initial solid matrix and decreased the porosity. We demonstrate that since the drying occurred through the top surface, the deposit is not uniform as a function of depth. Indeed, the particles are advected by the liquid flow toward the evaporative surface; the deposit is significant over a depth that depends on the initial volume fraction, but the pore size is affected over a very narrow length. These findings are interpreted in the frame of a physical model. This study may help to design better porous media and take into account particle influence in drying processes.

## 2. Materials and Methods

### 2.1. Materials

The fluid used in this study was an aqueous suspension of silica nanoparticles (Ludox HS40 from Sigma Aldrich). The initial volume fraction of particles is φ=24%. We also used a diluted suspension at a volume fraction of φ=5%; the suspension was obtained by dilution of the initial one with deionized water. The silica nanoparticles are hard and measured around a = 20 nanometers, with a density of 2.4. The particles increase the viscosity of the suspension; for φ=5%, viscosity is close to the one of pure water, at μ = 1.3 mPa.s; for φ=24%, the viscosity increase by a factor of 10, at μ = 15.4 mPa.s.

The porous media was a granular packing. Limestone sand (Carriere du Boulonnais) was sieved between 125 and 400 micrometers, with a density of 2.67. Its water absorption is 0.52%, which was neglected in this study.

The sand was packed manually over a height of H = 4 cm in a plastic syringe, closed at the bottom by an epoxy plug. The internal diameter was 9 mm. The sand was saturated with liquid. Tomography scans of the full sample (resolution of 7.5 micrometers) lasted more than 12 h, and generated data were hundreds of gigabytes; thus, only one sample for each volume fraction of silica nanoparticles was performed. In this paper, the two samples will be referred to by their initial particle volume fraction, φ=24% and φ=5%. The bulk porosity was calculated based on the mass and the density of each component. For φ=24%, the bulk porosity is 49%, and the initial water mass is 1.05 g; for φ=5% the bulk porosity is 57%, and the initial water mass is 1.16 g.

### 2.2. Drying Test

The cylindrical samples were glued to a holder and kept vertical. Water was allowed to evaporate only from the top surface. The samples were kept in a drying tunnel (cross section: 50 cm × 70 cm) where dried air was blown at a constant flow rate of 60 L/min [45,46]. The room temperature was kept at T=22 ± 1 °C. The mass was recorded automatically every 17 s. The drying rate was computed on smoothed data.

### 2.3. X-ray Microtomography

**Figure 1:**
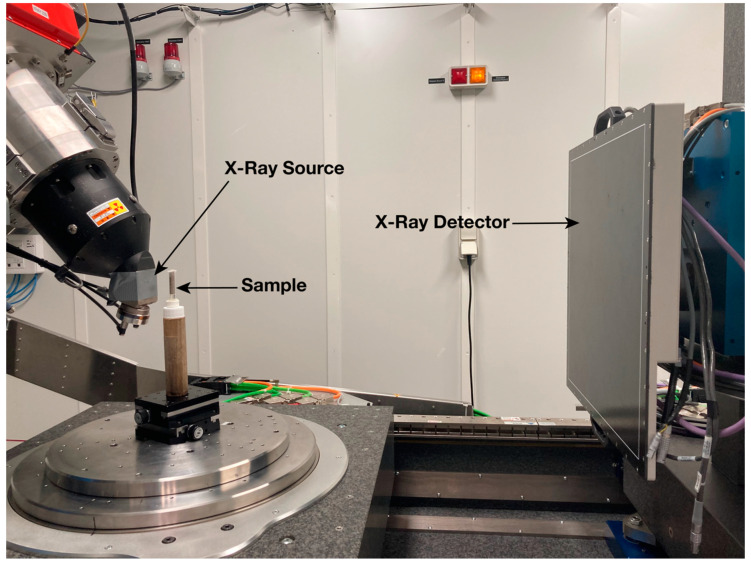
Picture of the X-ray microtomograph setup.

After drying, the samples were observed by X-ray tomography, which provides 3D images based on X-ray attenuation at the micron scale. Thus, the porous media could be observed non-destructively. The X-ray laboratory scanner Ultratom from RX-Solution (Annecy, France) was used, with a tension of 100 kV and a current of 50 µA for the sample prepared with φ=24%, and 34 µA for the sample with φ=5% (Figure 1). An X-ray detector (2992 × 2992 pixels) recorded 16 bits radiographs at a frame rate of 1 per second. To reconstruct the 3D images, samples were rotated over 360° by 2336 steps. For each step, eight radiographs were averaged to reduce noise. The voxel size was 7.5 micrometers. To observe the full sample, scans were performed at different heights. Four scans were realized for φ=24% and three scans for φ=5%. Thus, the 3D images of φ=24% covered the full sample, whereas only the top 2 cm for sample φ=5% was reconstructed. The total scanning time was 28 h. The experiments required computer storage of 240 gigabytes.

### 2.4. Scanning Electron Microscopy

The samples were cut in slices perpendicular to the principal axis of the cylinder. The exposed surfaces were observed by scanning electron microscopy (SEM). The SEM used in this study was a Hitachi TM4000Plus (Japan) in back-scattered electron mode. The vacuum was low to avoid charges accumulation in the non-conductive materials. The tension was set to 15 kV. The magnifications were set at X50 and X250 to compare various positions and between the two samples.

## 3. Results

The porous media initially saturated by a suspension of nanoparticles were monitored by mass loss during the evaporation process. Then, the final microstructures were investigated focusing on the particle deposits and their influence on the porosity.

### 3.1. Drying Kinetics

**Figure 2:**
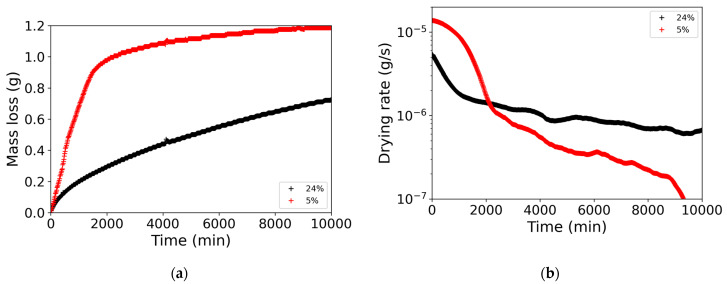
(**a**) Mass loss as a function of time; (**b**) drying rate as a function of time. Black symbols: initial particle volume fraction φ=24%; red symbols: initial particle volume fraction φ=5%.

Drying conditions were similar; even if perfect repeatability was never achieved, the drying conditions were constant during the experiments [37,47]. For initial particle volume fraction φ=24%, water dried slowly; indeed, the sample was not dried after one week, when 75% of the water had evaporated. For φ=5%, all the water had been evaporated after seven days. In fact, 75% of the water evaporated in the first 2000 min (1.5 days) (Figure 2a).

The initial drying rates were around $10^{-5}$ g/s. For φ=24%, the drying rate decreased immediately to $2\cdot 10^{-6}$ g/s. Then, after 1000 min, the drying rate shifted slowly down but stayed around $10^{-6}$ g/s. For φ=5%, initially, the drying rate remained high for 1000 min. Then, it decreased sharply by a factor of 10. Between 2000 and 9000 min, the drying rate constantly decreased, eventually decreasing by another decade as the sample dried out (Figure 2b).

.

### 3.2. Local Observations

After drying, the samples contained sand packing and dried nanoparticles. The particles formed a deposit in the initial porous media. To observe this deposit by SEM, samples were cut into slices. Initially, the sand packing was not consolidated and may have separated into pieces; but after drying with nanoparticles, the sand packing gained cohesion, and the first 2 cm could withstand the preparation process. The 2 cm of the bottom sample broke into grains as the samples were removed from molds. Thus, only the top half of the samples were observed by SEM.

For φ=24%, the top surface was covered by a crust of nanoparticles (see Table 1, first column). Indeed, as the sample was prepared, the sand packing settled, and some liquid bled. The pattern of the dried suspension showed cracks typical of paste and hard particle suspension [25,48]. Below the surface, the sand grains could be easily distinguished visually from the nanoparticle deposit, as the deposit was smooth with cracks. At 1 mm depth, the deposit seemed to fill the space between grains, leaving little porosity. At 15 mm depth below the free surface, the deposit was still clearly identified. The deposit covered large surfaces of the grains, but some large pores remained empty.

For φ=5%, grains were observed from the top surface, along with high porosity, compared to the crust of the other sample. At 1 mm depth, the nanoparticle deposit distinguished clearly visually from the grains. The deposit covered some grains and partially filled voids. However, the porosity could be identified as being a slight reduction in the initial porosity. At 15 mm depth, the deposit was not observed on the SEM even if the sand grains were consolidated. An energy dispersive X-ray analysis (EDX) analysis of the contact points (data not shown) showed that Silica was present at a high concentration. This suggests that the deposit should reinforce the contact point between grains. However, the porosity should have remained close to the initial porosity, as the deposit volume was negligible.

### 3.3. Modifications of the Porous Structure

As the samples were fully dried, we observed them by a non-destructive means: by X-ray tomography. This technique allowed us to obtain a full 3D image without demolding the samples from their tube. The direct observation of the images was quite similar to SEM images, even if the preparation did not alter the sample. The grayscale was based on X-ray attenuation, which is linked to density and atomic number. Thus, the voids were clearly identified from the porous matrix. Then, we could quantify the porous structure by computing the local porosity volume and size.

Indeed, the observation of the X-ray images confirmed that the SEM preparation did not significantly modify the porous media. Here, the images were not on fractured surfaces, but they were orthogonal slices in the undisturbed samples.

For φ=24%, we observed the cracked crust at the top surface (Table 2, left column). It did not contain grains but only nanoparticles deposit. At 1 mm depth, the grains were white, as calcium absorbs more X-ray than Silica (gray). Few voids (black) remained in the samples, but the nanoparticle deposit filled most spaces between grains. Similar to the top surface, the deposit was cracked, leaving some smaller voids in the porous media. At 15 mm depth, the deposit filled only a portion of the space between the grains, but a large portion of this space was porosity.

For φ=5%, the top surface contained grains, and the spaces were mostly filled with deposits (Table 2, right column). It was similar to the image for φ=24% at 1 mm depth. For φ=5%, at 1 mm depth, a significant part of the space between grains was left empty; at 15 mm depth, the deposit could not be observed, and the sand packing was filled by air.

**Figure 3:**
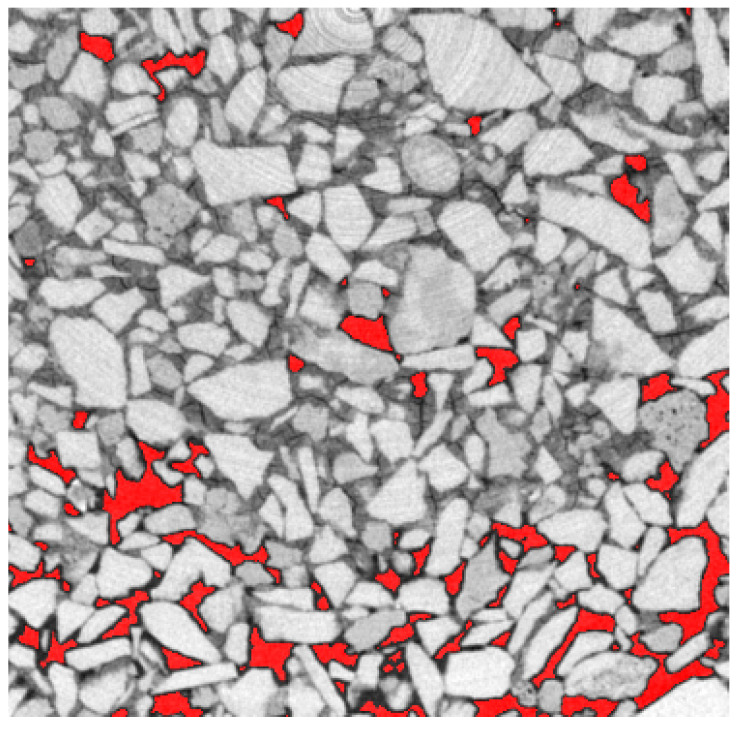
Microtomography image with the identified porosity in red, for φ=5% at 1 mm depth.

To compute the porosity, the voxel with air should be identified on the 3D images. A Gaussian blur was applied to smooth the images and reduce the noise on the gray values. Then, far from the surface, where air represents a large fraction of the porosity, a threshold of gray value was identified to isolate the air voxel from the porous matrix (grains + deposit). This simple approach has many limitations, such as the influence of the image resolution (here 7.5 microns) or the porosity at the boundary with the matrix; indeed, some voxels were not in a pure phase. Nevertheless, these approximations should impact all cross sections. Thus, we considered that the global trends present here should hold even if the absolute value shifts slightly with a deeper analysis of the data (Figure 3).

**Figure 4:**
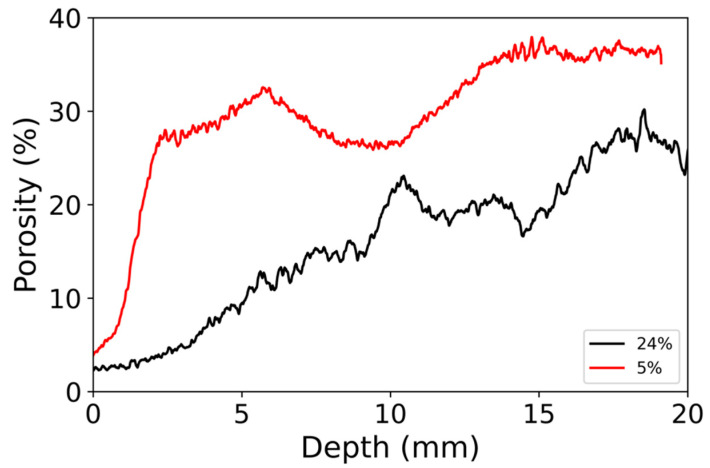
Porosity profile as a function of depth from the top surface. Black: initial particle volume fraction φ=24%; red: initial particle volume fraction φ=5%.

For φ=24%, the porosity far from the surface was around 25%, even though it fluctuated from 20 to 30% (Figure 4). These strong variations may be due to hand packing. Closer to the top surface, the porosity decreased constantly, down to 2% at the top surface. Indeed, the top surface was identified as the position of the minimum porosity. Given the strong fluctuation of the porosity, it was difficult to define a bottom part where porosity would be the initial one; we considered that porosity was reduced from its initial value in the first 15 mm.

For φ=5%, the porosity was significantly larger than for the other sample; the porosity away from the surface ranged between 25 and 37% (Figure 4). The porosity was strongly reduced in a very thin layer of 2 mm. At the top surface, the porosity was only 5%, and it increased sharply to 27% at 2 mm depth. Below 2 mm, the porosity did not have a clear trend. The porosity was higher for φ=5% than for φ=24%, even in the area without particle deposit; thus, we suggest that this is due to variations in the initial granular packing.

**Figure 5:**
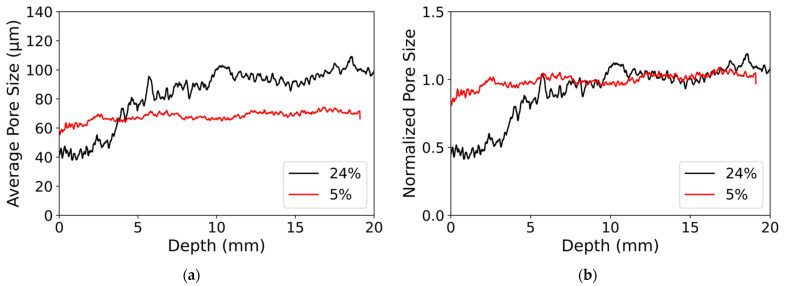
(**a**) Average pore size, in a slice perpendicular to the main axis of the cylindrical sample, as a function of depth from the top surface; (**b**) average pore size normalized as a function of depth from the top surface. Black lines: initial particle volume fraction φ=24%; red lines: initial particle volume fraction φ=5%.

From the threshold images, the pore size was also computed. The pore size at a given voxel corresponds to the largest sphere inserted in the air phase containing this particular voxel. This very computer-intensive computation was performed over all voxels in the porosity through an ImageJ function “Local Thickness” [49]. For each slice, perpendicular to the main axis of the cylindrical sample, the average pore size was computed (Figure 5).

For φ=24%, the average pore size varied as a function of the depth from the top surface. In the first 4 mm, the pore size was around 40 µm, then it increased sharply in a transition zone between 4 and 5 mm depth, and it reached a plateau at 90 µm (Figure 5a).

For φ=5%, the pore size was uniform in the sample at 60 µm. The pore size in the area unaffected by the deposit was slightly smaller than for the other sample. This may be due to the hand packing of the sample or variation in the sand particle size distribution inside the batch. However, the average pore size difference was less of interest in this study than the variation. Thus, Figure 5b shows the pore size normalized by the plateau value. The difference in pore size between the two samples appeared to be located in the first 5 mm.

## 4. Discussion

To understand the heterogeneous deposition of the nanoparticles, we first discuss the drying process and its coupling with nanoparticle deposition. Finally, we examine the consequences on the permeability of the modified porous media.

### 4.1. Advection of Nanoparticles

As water evaporates from the free surface, it is partially replaced by water flowing from the samples [13,36,37]. Indeed, the general process of drying is driven by capillary forces [8,9,10,11]. In most permeable porous media and without gravity effects, the sample remains uniformly wet owing to the capillary effect [12,13]. The evaporated water at the free surface is replaced by liquid flow. The velocity of evaporation $V_{e}$, expressed as a liquid flow, is computed from the measured drying rate as follows:

(1) $$V_{e}=\frac{-1}{\rho_{0}S}\cdot \frac{dm}{dt},\;$$

where $S=6.3\cdot 10^{-5}$ m^2^ is the surface area, $\rho_{0}$ is the fluid density and dm/dt is the drying rate. In our experiments, the water evaporates mainly at a drying rate between $dm/dt=10^{-5}-10^{-6}$ g/s. Additionally, the fluid density is $\rho_{0}=1260$ kg/m^3^ for φ=24%, and $\rho_{0}=1050$ kg/m^3^ for φ=5%. Thus, the evaporation velocity scales within the range of $V_{e}=10^{-4}-10^{-5}$ m/s.

As water evaporation creates a liquid flow towards the free surface, the suspended nanoparticles are transported and should accumulate at the surface. Nevertheless, this process may be counterbalanced by diffusion, which tends to equilibrate the nanoparticle concentration in the fluid. The diffusion coefficient could be estimated based on Stokes–Einstein diffusion.

(2) $$D=\frac{k_{B}T}{6\pi \mu a},$$

where $k_{B}$ is the Boltzmann’s constant. The comparison between advection and diffusion is given by the dimensionless Peclet number as follows:

(3) $$Pe=\frac{V_{e}H}{D},$$

where H is the sample height, as the flow replaced water at the surface by water uniformly distributed in the sample. Here, $Pe=5\cdot 10^{4}-10^{5}$; thus, the nanoparticles are advected by the fluid towards the evaporative surface. As the water dries, the nanoparticles are left to form a solid deposit.

### 4.2. Gradient of Nanoparticle Deposit

The deposit of nanoparticles depends strongly on the liquid saturation with time. As saturation decreases, deposits will accumulate in a smaller fraction of the porosity. Indeed, in the beginning, the pores are saturated with liquid, and the nanoparticles accumulated at the top surface of the sample. Then, the liquid receded and left a dried area at the top; the particles accumulated further away from the surface. This process is described in more detail in [36]. In particular, the receding front of liquid leads to a slower drying rate, as water diffuses over a greater length [37]. Thus, the drying rate is not constant but decreases slightly for φ=5% and significantly for φ=24% (Figure 2).

Based on this previous study [36], validated on glass bead packings, the porosity is reduced mostly at the free surface and the final porosity ϕ profile is

(4) $$\phi =\phi_{0}\left[1-\varphi_{m}{\left(1-\frac{z}{H}\right)}^{\left(\varphi_{m}-\varphi \right)/\varphi }\right],\;$$

where $\phi_{0}$ is the initial porosity, and $\varphi_{m}=60\%$, is the packing fraction of nanoparticles. This model shows that for φ=5%, the porosity is decreased from its initial value in the first centimeter and that the full sample is affected for φ=24%. Indeed, this porosity profile is an oversimplification that could not be directly compared to our measurement of Figure 3 because the initial porosity had strong fluctuations. However, the general process is similar, and the porosity is affected over a very narrow depth close to the surface for φ=5%. For φ=24%, the porosity gradient extends over half the sample.

For φ=24%, the pore size decreased over a smaller length than the porosity and the pore size was smaller than in the bottom part in the first 5 mm; for φ=5%, the pore size is uniform as a function of depth. Indeed, a large quantity of nanoparticle deposit is needed to reduce the pore size. SEM images suggest that the small deposit volume only covered the roughness of the grains, and it did not modify the pore size. However, at a large deposit volume, it clogged the large space between grains (see Table 1).

Thus, the main feature of particles deposit could be summarized as follows: the pore size decreased significantly over a very restricted depth. However, the pore size decreased over a larger depth, extending up to the full sample size depending on the initial volume fraction of suspended particles.

Based on Equation (4), to measure the transport of particles, the study presented in [36] defined a characteristic length over which the deposit is significant, compared to the initial volume fraction of nanoparticles in the fluid φ. However, from the porous media point of view, it may be more meaningful to define a length over which the porosity is decreased to $0.8\cdot \phi_{0}$, compared to the initial porosity $\phi_{0}$. Thus, we defined this length λ as

(5) $$\lambda =H\left[1-{\left(0.2\cdot \varphi_{m}\right)}^{\varphi /\left(\varphi_{m}-\varphi \right)}\right].$$

For φ=5%, we found λ=0.7 cm, and for φ=24%, λ=3 cm. As they are based on a very simple model, these values overestimated the length over which the porosity was significantly reduced in our experiments (see Figure 3).

## 5. Conclusions

Dried porous media initially saturated by nanoparticle suspensions were observed by SEM and X-ray microtomography. As the suspensions were fully dried, nanoparticles formed a solid deposit, which added to the initial solid matrix and decreased the porosity. Particles were advected and transported close to the free surface by the water flow due to drying.

The receding evaporation front slowed down the drying rate, and the gradient of particles appeared even if the heterogeneous structure of the porous media made it challenging to capture this effect with a simple model. The pore size was reduced in a very narrow length, but the porosity decreased significantly over a characteristic length depending on the initial particle volume fraction, which was quantified.

The consequences of the deposit will affect the porous media transfer properties. Mostly, the permeability may be significantly reduced by the decrease in pore size and porosity. This may be important to protect the porous structure from aggressive agents transported by fluids, such as chloride attacks in cement. On the contrary, soils will be less permeable to the water needed to feed plants for agriculture.

## Figures and Tables

**Table 1:** SEM image of the dried samples at three depths from the top drying surface. Left: initial particle volume fraction φ=24%; right: initial particle volume fraction φ=5%.

Depth	φ=24%	φ=5%
Top surface	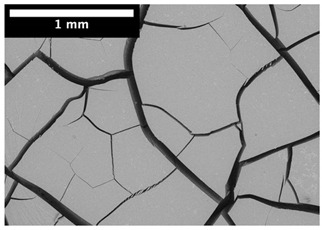	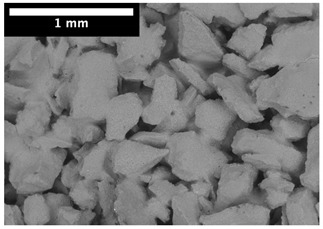
1 mm	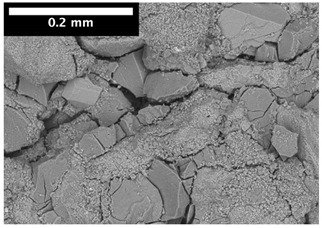	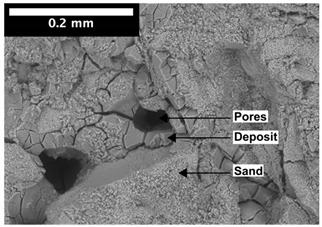
15 mm	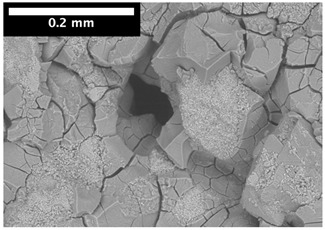	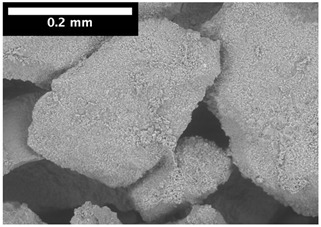

**Table 2:** Microtomography images, perpendicular to the main axis of the cylindrical samples, of the dried porous media with nanoparticle deposits. Left column: initial particle volume fraction φ=24%; right column: initial particle volume fraction φ=5%. All images have the same resolution, and the scale bar on the first image applies to all images.

Depth	φ=24%	φ=5%
Top surface	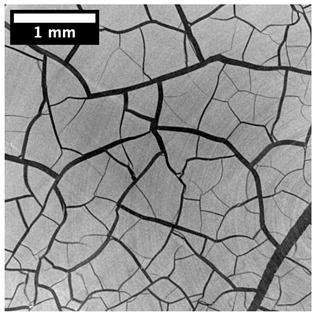	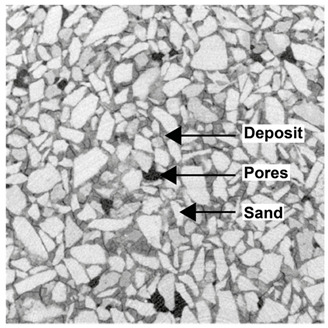
1 mm	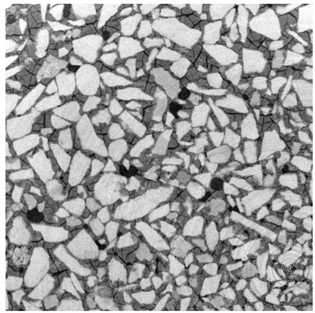	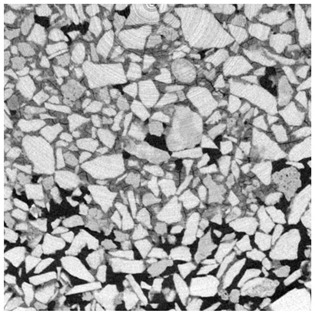
15 mm	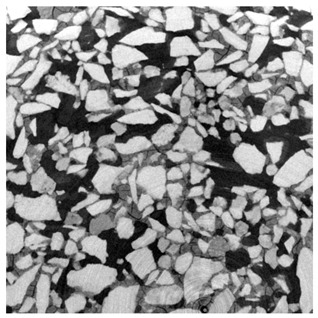	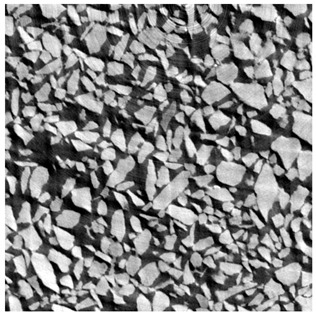

## Data Availability

Not applicable.
